# Does the critical shoulder angle decrease after acromioplasty? A systematic review and meta-analysis

**DOI:** 10.1186/s13018-022-02927-7

**Published:** 2022-01-15

**Authors:** Mingtao Zhang, Zhitao Yang, Borong Zhang, Tao Liu, Jin Jiang, Xiangdong Yun

**Affiliations:** Department of Orthopaedics, Lanzhou University Second Hospital, No. 82 Cuiyingmen, Chengguan District, Lanzhou, 730030 Gansu China

**Keywords:** Critical shoulder angle, Acromioplasty, Systematic review

## Abstract

**Background:**

Rotator cuff tears are one of the most common shoulder injuries in the older population. This study aimed to determine whether acromioplasty reliably decreases the critical shoulder angle (CSA) and describe any associated complications.

**Methods:**

A systematic literature review was performed according to PRISMA guidelines using PubMed, EMBASE, Web of Science, and Cochrane Library Database. Two reviewers independently screened the titles and abstracts using prespecified criteria. Studies where the acromioplasty was performed as a surgical procedure were included. Patient characteristics and degree of CSA reduction were collected from each individual study. All statistical analyses were performed using Review Manager (RevMan) 5.4.1 software. A random-effects model was used for meta-analysis.

**Results:**

A total of 9 studies involving 1236 patients were included in the meta-analysis. The age of patients ranged from 23 to 82 years. The follow-up period ranged from 12 to 30 months. Of the 9 studies, 8 (88.9%) were retrospective, 1 (11.1%) was prospective, 5 were comparative, and 4 were case series. The mean CSA was significantly reduced from 36.1° ± 4.6° to 33.7° ± 4.2 (*p* < 0.05). The meta-analysis showed an overall best estimate of the mean difference in pre- and postoperative CSA equal to 2.63° (95% confidence interval: 2.15, 3.11] (*p* < 0.00001).

**Conclusions:**

Acromioplasty can significantly reduce CSA, notably in cases of high preoperative CSA. In addition, the effect of lateral acromioplasty on the CSA was more significant compared to anterolateral acromioplasty. Acromioplasty was not associated with complications during the short-term follow-up.

**Supplementary Information:**

The online version contains supplementary material available at 10.1186/s13018-022-02927-7.

## Introduction

Rotator cuff tears (RCTs) are one of the most common shoulder injuries in the general older population [1, 2]. Among people aged > 60 years, the incidence of RCTs is estimated at > 10% [3]. While the pathogenesis of degenerative RCTs is multifaceted, the precise mechanisms are still unclear [4]. RCTs result from numerous risk factors, including intrinsic and extrinsic factors. The extrinsic risk factors, especially acromial morphology, have attracted the attention of many scholars. Numerous studies have reported that a higher acromion index, as well as type III and lower lateral acromion angles, are significantly related to degenerative RCTs [5–7].

**Fig. 1:**
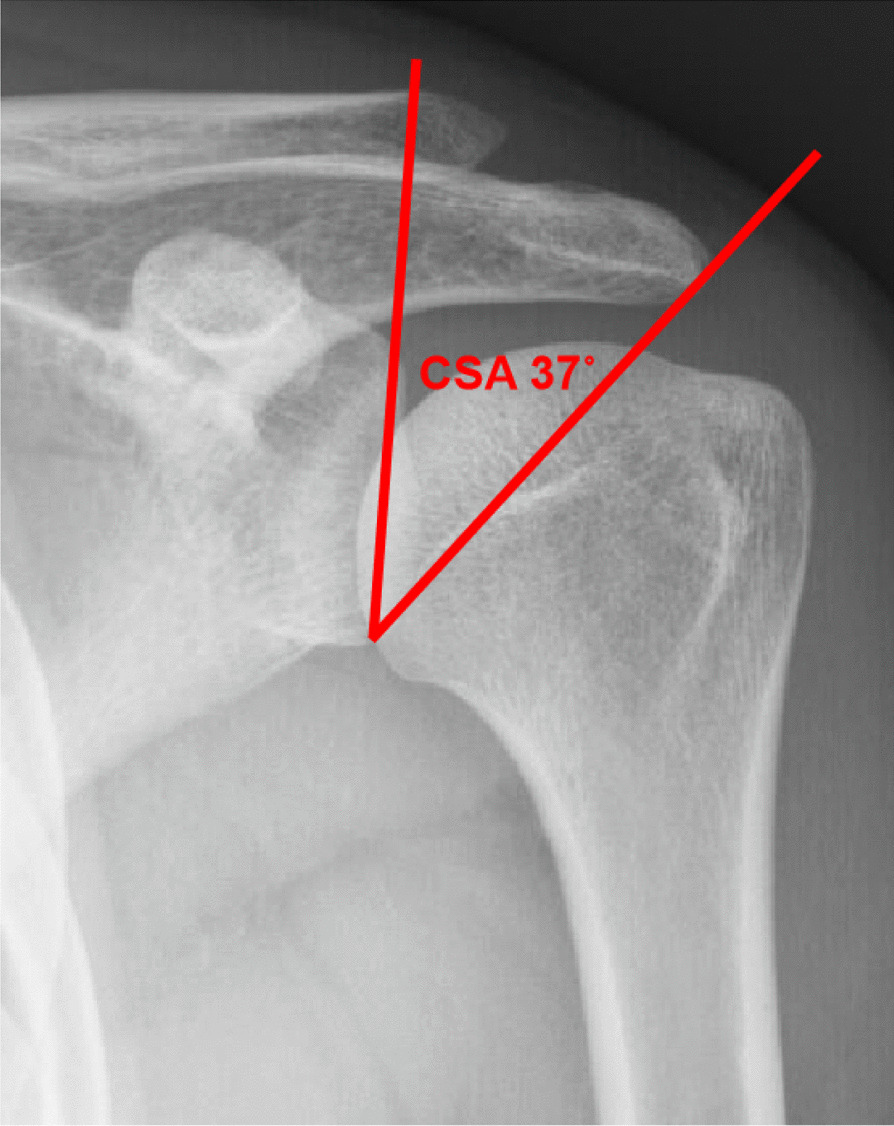
The critical shoulder angle (CSA) measured on true anteroposterior radiographs

The correlation between degenerative RCTs and the CSA has recently received increasing attention [8, 9]. The CSA, first mentioned in RCT research in 2013 by Moor et al. [10], is defined as the angle between the superior and inferior bony margin of the glenoid and the lateral margin of the acromion (Fig. 1). The CSA can be quantified by standard radiographic imaging of the shoulder. Moor et al. [10] reported that patients with CSAs > 35° had a higher rotator cuff tear rate compared to those with CSAs ≤ 35°. In addition, a recent study found that CSAs > 38° postoperatively increased a patient's risk of rotator cuff retear in 14-fold [11]. Gerber et al. [12] found that patients with CSAs > 35° had a higher retear rate after repair than those with CSA < 33° after lateral acromioplasty. However, a recent meta-analysis showed that it was difficult to obtain an exact association between the CSA and degenerative rotator cuff tears [13].

Acromioplasty was originally described in 1972 by Neer et al. [14] to solve the anteroinferior bone impingement of the rotator cuff tendon. Ellman et al. [15] were the first to establish the principles of arthroscopic acromioplasty in 1987. Some studies have demonstrated that anterolateral or lateral acromioplasty can significantly reduce the CSA [12, 16]. Katthagen et al. [17] demonstrated that the CSA can be reduced by arthroscopic anterolateral or lateral acromioplasty in a cadaveric study. In addition, Kaiser et al. [18] reported that lateral acromioplasty reduced the CSA more significantly than anterolateral acromioplasty in an anatomical study. However, Olmos et al. [19] demonstrated that the CSA cannot always be reduced to < 35° by arthroscopic lateral acromioplasty, especially in patients with preoperative CSAs > 40°.

To clarify the available evidence, the purpose of this systematic review and meta-analysis was to determine if acromioplasty reliably decreases the CSA and determine any association with postoperative complications.

## Methods

The PRISMA (Preferred Reporting Items for Systematic Reviews and Meta-Analyses) guidelines were implemented when conducting and reporting this review and meta-analysis [20]. PRISMA checklist were showed in Additional file 1.

### Literature search

We consulted an independent information specialist during the design phase of the search process. A search strategy was developed and performed using PubMed, EMBASE, Web of Science, and Cochrane Library Database up to August 2021 for all English-language publications. The following search terms were used: (“Acromioplasty” OR “Acromion”) AND (“Critical shoulder angle” OR “CSA”). We identified potential articles by screening titles and abstracts, and if these meet the inclusion criteria, the full text of the article was obtained. The reference lists from the included articles were analyzed to identify other additional articles.

### Inclusion and exclusion criteria

Studies which met the following inclusion criteria were reviewed for inclusion: (1) clinical trial investigating patients with degenerative RCTs treated by acromioplasty; (2) studies that reported any outcomes, including functional scores, patient-reported outcomes (PROs), and change of CSA; and (3) English-language studies. The exclusion criteria were: (1) animal studies; (2) case reports, review articles, meta-analysis, technical notes, abstract-only articles, and biomechanical studies; (3) studies with missing data on clinical outcomes; and (4) non-English language.

### Assessment of study quality

The Methodological Index for Non-Randomized Studies (MINORS) score was used by two reviewers to evaluate the quality of all included articles [21]. A score of 0 (not reported), 1 (reported but inadequate) or 2 (reported and adequate) was given for each of the 12 items on the MINORS scoring system with a maximum score of 16 for non-comparative studies and 24 for comparative studies. Methodological quality was categorized a priori as follows: a score of 0–8 or 0–12 was considered poor, 9–12 or 13–18 was considered intermediate, and 13–16 or 19–24 was considered high for non-comparative and comparative studies, respectively. The level of evidence was reported based on the criteria accepted by the American Academy of Orthopaedic Surgeons [22]. Any disagreements between the two reviewers were resolved by consensus after discussion.

### Data extraction

Two reviewers independently selected suitable articles for full-text review by screening all titles and abstracts. Data from the included articles were extracted, including authors, publication date, sample size, level of evidence, patient demographics, study design, and CSA thresholds. If these data were not provided, we contacted the authors directly. Studies were excluded from further analysis when the author could not provide the missing data.

### Statistical analysis

Data from all studies were extracted and tabulated to show the degree of CSA reduction after acromioplasty, repair technique, and CSA thresholds. The primary outcome was the change of CSA after acromioplasty in patients with larger CSAs. A forest plot of the comparative studies was prepared using Review Manager software (RevMan) v.5.4.1, 2020 (The Cochrane Collaboration, Copenhagen, Denmark). The *I*^2^ statistic was used to assess heterogeneity. An *I*^2^ ≤ 50% was considered a slight statistical heterogeneity among studies, and a fixed-effect model was used for analysis. For *I*^2^ > 50%, the random-effect model was used for analysis.

## Results

### Literature search

**Fig. 2:**
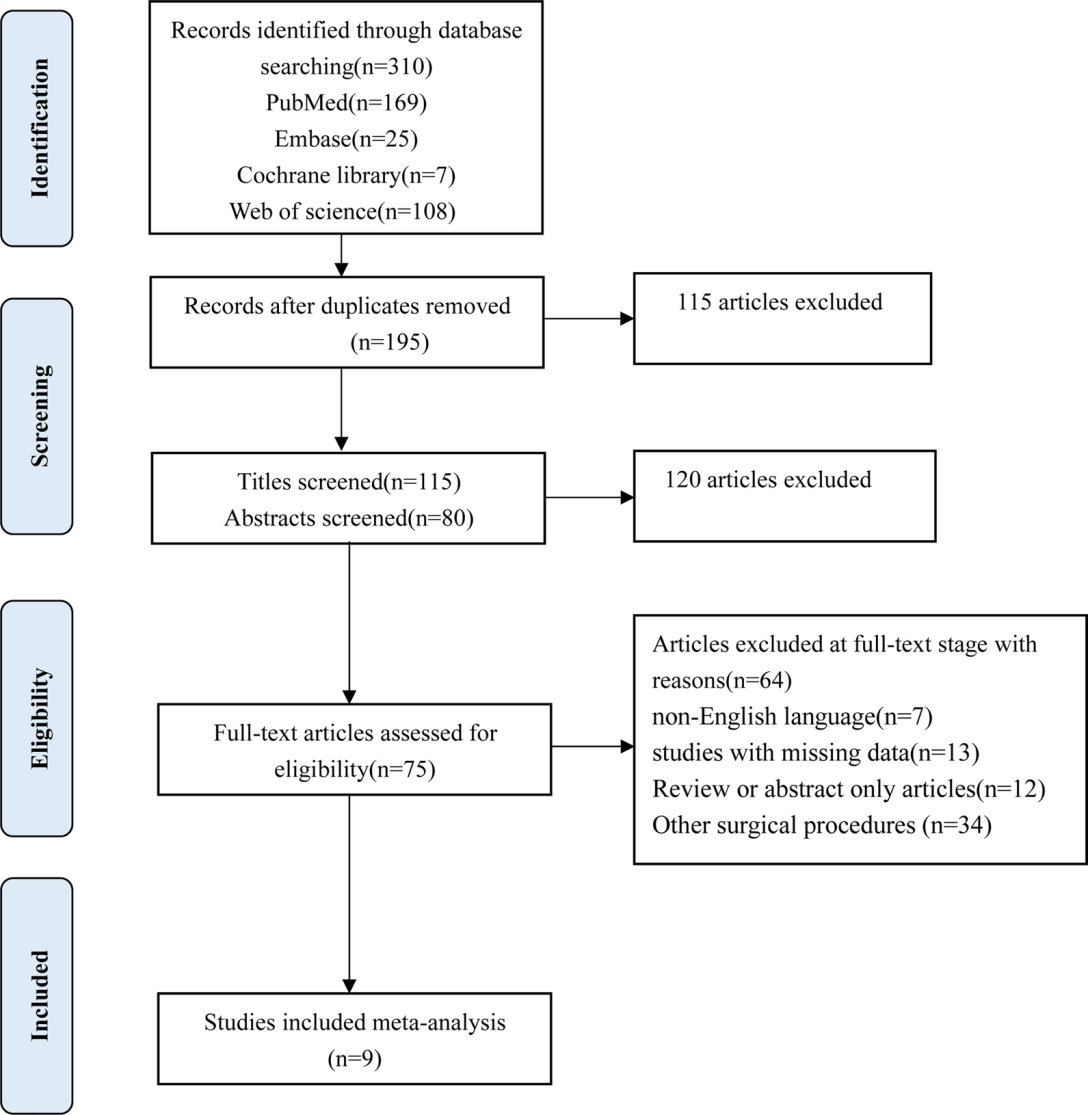
Search result (flow diagram)

A total of 310 unique studies were identified for review. Most of the studies were excluded as they did not meet the inclusion criteria. In total, 75 articles were potentially suitable after the title and abstract screening. From the full-text assessment, 9 articles with 1236 patients met the inclusion criteria. A flowchart of the literature search is provided in Fig. 2.

### Patient and study characteristics

A total of 9 studies involving 1236 patients were included in the meta-analysis with ages ranging from 23 to 82 years. The follow-up period ranged from 12 to 30 months. Of the 9 studies, 8 (88.9%) were retrospective, 1 (11.1%) was prospective, 5 were comparative, and 4 were case series. Of the included studies, 6 were conducted in Europe (3 in France, 2 in Switzerland, and 1 in Italy), 2 in Asia (1 in China and 1 in Taiwan), and 1 in North America (1 in the United States). The publication year of the articles ranged from 2015 to 2021 with 7 studies after 2020. There were 1 Level II, 4 Level III, and 4 Level IV studies. Based on the MINORS criteria (Table 1), the mean study quality score was 16.3 ± 4.5. For the comparative studies, the mean MINORS score was 19.4 ± 1.5 out of 24. For the 4 non-comparative studies, the mean MINORS score was 11.3 ± 0.5 out of 16. A total of four high-quality studies, and a further seven intermediate-quality studies were identified. The study characteristics and patient demographics are shown in Table 2.

**Table 1:** Quality assessment of included studies

Authors	Clearly stated aim	Inclusion of consecutive patient	Prospective collection of data	Endpoints appropriate for aim	Unbiased assessment of endpoints	Appropriate follow-up period	Lost to follow-up < 5%	Prospective calculation of study size	Adequate control group	Contemporary groups	Baseline equivalence of groups	Adequate statistical analysis	Total score
Gerber (2017)	2	2	0	2	2	2	2	0	0	0	0	0	12
Billaud (2019)	2	2	0	2	2	1	2	0	0	0	0	0	11
Franceschetti (2020)	2	2	0	2	2	2	2	0	2	2	2	1	19
Long (2020)	2	2	2	2	1	2	2	2	2	2	1	2	22
Olmos (2020)	2	2	0	2	2	2	2	0	2	2	1	1	19
Girard (2020)	2	2	0	2	2	1	2	0	0	0	0	0	11
MacLean (2020)	2	2	0	1	2	1	2	0	2	2	2	2	18
Lin (2021)	2	2	0	2	2	1	2	0	2	2	2	2	19
Hardy (2020)	2	2	0	2	2	1	2	0	0	0	0	0	11

**Table 2:** Characteristics of included studies

Lead author (year)	Location	Total participant	Age (years)	Gender (male%)	Follow-up (months)	Study design (level of evidence)	Measurement method of CSA	Repair Technique	**CSA threshold**
Gerber (2017)	Switzerland	49	39–76	83.70%	30	Case Series(IV)	MRI	Arthroscopic lateral AP	NR
Billaud (2019)	France	90	41–76	61.10%	NR	Case Series(IV)	Radiographs	Arthroscopic anterior AP	NR
Franceschetti (2020)	Italy	289	57	46.70%	28	Retrospective comparative study(III)	Radiographs	Arthroscopic lateral AP	35
Long (2020)	China	60	NR	NR	12	Prospective comparative study(II)	3D-CT	Arthroscopic lateral and anterolateral AP	33
Olmos (2020)	France	90	58	60%	12	Retrospective comparative study(III)	Radiographs	Arthroscopic lateral AP	35
Girard (2020)	France	148	29–80	57.40%	NR	Case Series(IV)	Radiographs	Open anterior AP	35
MacLean (2020)	USA	71	58	64.80%	NR	Retrospective comparative study(III)	Radiographs	Arthroscopic lateral AP	35
Lin (2021)	Taiwan	337	64.2	47.50%	NR	Retrospective comparative study(III)	Radiographs	Arthroscopic anterolateral AP	38
Hardy (2020)	France	102	23–82	37.20%	NR	Case Series(IV)	Radiographs	Arthroscopic lateral AP	35

NR, not reported; CSA, critical shoulder angle; AP, acromioplasty; MRI, magnetic resonance imaging; 3D-CT, three-dimensional computerized tomography

### Surgical procedures

Eight studies described the technique used for arthroscopic acromioplasty. In total, 1 study described open acromioplasty, 5 lateral acromioplasty, 2 anterior acromioplasty, 1 anterolateral acromioplasty, and 1 lateral and anterolateral acromioplasty (Table 2).

### Meta-analysis

**Fig. 3:**
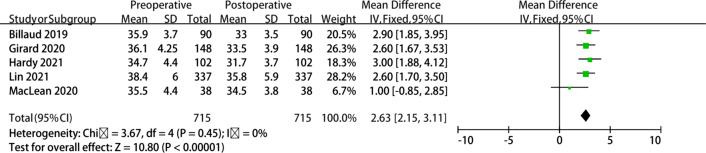
The forest plot of acromioplasty studies

After excluding 4 studies that did not include the mean plus SD of the CSA, a total of 5 studies including 715 patients were used to evaluate acromioplasty outcomes (Table 3). The mean CSA was significantly reduced from 36.1° ± 4.6 to 33.7° ± 4.2 (*p* < 0.05) with a significant decrease in the postoperative compared with the preoperative CSA mean. The mean differences were investigated to determine the overall best estimate of 2.63 (95% confidence interval (CI) 2.15, 3.11; *I*^2^ = 0%), measured in SD units of the difference in scores (*p* < 0.00001). A forest plot of the paired standardized mean differences is shown in Fig. 3.

**Table 3:** The reduction in CSA after acromioplasty

Study	Preoperative			Postoperative		
	Mean	SD	Total	Mean	SD	Total
Billaud (2019)	35.9	3.7	90	33	3.5	90
Girard (2020)	36.1	4.25	148	33.5	3.9	148
MacLean (2020)	35.5	4.4	38	34.5	3.8	38
Hardy (2021)	34.7	4.4	102	31.7	3.7	102
Lin (2021)	38.4	6	337	35.8	5.9	337

CSA, critical shoulder angle; SD, standard deviation

#### Lateral acromioplasty

Lateral acromioplasty was reported in 5 studies. Gerber et al. [12] reviewed 49 consecutive patients and found that the mean CSA decreased from 37.5° preoperatively (95% CI 36.7°, 38.3°) to 33.9° postoperatively (95% CI 33.3°, 34.6°; *p* < 0.001). Franceschetti et al. [23] reported a positive effect in patients with a CSA > 35° after lateral acromioplasty. A total of 2 studies reported that the mean CSA was reduced by lateral acromioplasty. In addition, Olmos et al. [19] reported that when the preoperative CSA was > 40°, the respective postoperative CSA remained > 35° in 83.3% of cases (*p* < 0.001).

#### Anterior acromioplasty

There were 2 studies describing anterior acromioplasty to reduce the CSA. Billaud et al. [16] reported that the average CSA for patients preoperatively was 35.9° (± 3.7°; range: 26.2°–44.2°) and 33° after the anterior acromioplasty (± 3.5°; range: 24.8°–41.4°). Girard et al. [24] reported a mean preoperative CSA of 36.1° (± 4.25°; range: 25°–48.4°) and a postoperative CSA of 33.5° (± 3.9°; range: 23.8°–45.2°), for a significant decrease of − 2.6° ± 2.5° (*p* = 0.001).

#### Anterolateral acromioplasty

Anterolateral acromioplasty was described in 2 studies. Lin et al. [25] reported that 337 participants presented with a mean CSA of 38.4° ± 6.0° before surgery, which significantly decreased to 35.8° ± 5.9° after anterolateral acromioplasty (*p* < 0.05). Katthagen et al. [17] reported that anterolateral acromioplasty decreased the CSA by a mean of 1.4° (95% CI 0.8°, 1.9°).

#### Lateral versus anterolateral acromioplasty

One study reported that the effect on the CSA of lateral acromioplasty was more significant compared to anterolateral acromioplasty. Long et al. [26] found that the reduction in CSA was 2.6° ± 1.8° by anterolateral acromioplasty and 4.4° ± 1.5° by lateral acromioplasty. In addition, an anatomic cadaveric study reported that the mean preintervention CSA (34.3° ± 2.1°) decreased via anterolateral acromioplasty (33.1° ± 2.0°, *p* < 0.001) and further decreased by lateral acromioplasty (31.5° ± 1.7°, *p* < 0.001) [17]. An anatomical study [18] reported that lateral acromioplasty of 5 mm/10 mm reduced the CSA significantly more than anterolateral acromioplasty of 5 mm/10 mm [5 mm: 2.3° ± 0.8° vs. 1.2° ± 1.1°, *p* = 0.0002]/[10 mm: 4.8° ± 1.3° vs. 2.7° ± 1.7°, *p* = 0.0001].

### Functional outcomes and complications

The most universally used functional outcome measure was the Constant–Murley scores reported in 3 studies. One study reported that the scores had improved from 59 points (range, 54–64 points) preoperatively to 74 points (range, 70–78 points) postoperatively at a mean follow-up of 30 months (range, 12–47 months) [12]. Another study also found a significant improvement in Constant–Murley score after lateral acromioplasty [23]. No complications were reported in any of the included studies related to acromioplasty.

## Discussion

This systematic review and meta-analysis aimed to determine if acromioplasty reliably decreases the CSA and to describe any association with postoperative complications. Our main findings indicate that the acromioplasty procedure can effectively reduce the CSA, the effect of lateral acromioplasty on the CSA is more significant compared to anterolateral acromioplasty, and acromioplasty is not associated with complications based on short-term follow-up.

The CSA, originally introduced by Moor et al. [10], is thought to influence the risk of degenerative RCTs, with a range of 30° to 35° generally considered to be a “favorable range.” Studies have found that individuals with degenerative RCTs have significantly larger CSAs (≥ 35°) compared to those with asymptomatic shoulders and that a CSA < 30° is associated with glenohumeral osteoarthritis. Gerber et al. [27, 28] confirmed that a large CSA alters glenohumeral biomechanics such that could induce supraspinatus overload and that a low CSA increased the load of the humeral head on the glenoid. Furthermore, Garcia et al. [11] reported that contrary to previous studies, the average CSA values correlated with RCTs, where CSAs > 38° (range: 35°–39°) seemed to be a consistent predictor of RCTs and indicated an increased risk of retear after surgical repair. However, a meta-analysis showed that while the CSA can be reliably measured, the difference in the CSA between cases and controls varied from very large to almost no difference, and it is difficult to understand the strength and association between the CSA and RCT with the current evidence [13]. In addition, Cerciello et al. [29] confirmed no significant differences in CSA values between patients who had undergone shoulder replacement and experienced late cuff failure and those in whom the same procedure had been successful. Therefore, larger populations are needed to confirm this trend.

CSAs in partial-thickness tears and full-thickness tears were reported in some studies. Pandey et al. [30] reported that higher CSAs are associated with a full-thickness tear but not with partial tears. A meta-regression analysis revealed that the sensitivity of CSA could be higher for differentiating full-thickness RCTs and normal patients [9]. However, another study reported that the mean CSA in patients with full-thickness tears was 34.3 ± 4.2° and those with partial-thickness tears was 32.6 ± 3.2° (*p* = 0.08) [31]. Furthermore, Chalmers et al. [32] demonstrated that CSA is not correlated with tear size or progression and does not seem to change with time. Therefore, further studies are needed to clarify whether larger CSA is only associated with full-thickness tears.

The methods used to measure CSA mainly include radiographs, CT imaging, and magnetic resonance imaging (MRI). Spiegl et al. [33] reported that interobserver and intra-observer agreement on radiographs were 0.87 (95% CI 0.78, 0.93) and 0.91 (95% CI 0.82, 0.96), respectively. Therefore, they considered that CSA measurements obtained on radiographs demonstrated excellent interobserver agreement with less variability than CSA measurements by MRI, especially in osteoarthritis patients. Furthermore, Samy et al. [34] measured the CSA of 60 shoulders by radiographs and multiplanar reconstructions of corresponding CT scans and found that the measurements of the CSA on anterior–posterior radiographs and CT scans are highly correlated with negligible inter-modality differences. In the present analysis, radiographs were used in 7 studies, CT in 1 study, and MRI in 1 study.

There is no standard evaluation of acromial resection. Gerber et al. [12] reported that the mediolateral diameter of the acromion was reduced by an average of 6 mm (range, 3–8 mm) after lateral acromioplasty. In another anatomic cadaveric study, researchers demonstrated that a 5 mm lateral acromion resection reduced the CSA significantly and did not damage the deltoid origin [17]. In addition, Kaiser et al. [18] reported that lateral acromioplasty of 5/10 mm reduced the CSA significantly (5 mm: 2.3° ± 0.8° [range: 0.7°–3.6°] vs. 10 mm: 4.8° ± 1.3° [range: 2.1°–7°]). Although lateral acromioplasty up to 10 mm has been considered a safe technique [12, 17], other studies found that over-resection of the acromion has potential complications, including acromial fractures and detachment of the deltoid origin [35, 36].

Currently, altering the CSA by arthroscopic acromioplasty is a common strategy. Girard et al. [24] compared the effect between arthroscopy and open surgery and found that surgical technique did not affect change in CSA (open surgery: − 2.3° ± 1.9° [range: − 6.3° to − 1°] vs. arthroscopy: − 2.7° ± 2.7° [range: − 10.5° to − 5°]; *p* = 0.06). In the present systematic review, 8 studies described the techniques used for arthroscopic acromioplasty, and one studies described open acromioplasty. Therefore, arthroscopic acromioplasty was a useful and safe procedure.

There remains controversy regarding the usefulness of acromioplasty performed at the time of rotator cuff repair (RCR). Some studies have reported no difference in functional outcome scores for patients who underwent arthroscopic RCR with or without acromioplasty [37–39]. In addition, several studies concluded that the reduction in the CSA does not improve functional results postoperatively [40, 41]. However, some studies reported that a large CSA increased the risk of retearing after RCR [11, 42, 43]. Therefore, future studies are warranted to clarify the usefulness of acromioplasty at the time of RCR.

### Study strengths and limitations

This study has several strengths. First, it is the first systematic review to determine whether acromioplasty reliably decreases the CSA and if it is associated with complications. Second, we included all studies associated with acromioplasty aimed to reduce the CSA. Finally, we assessed the studies according to the PRISMA statement to improve rigor.

This systematic review also has some limitations. First, this systematic review included 9 studies, of which, 8 (88.9%) were retrospective and only 1 study (11.1%) was prospective. The retrospective studies were limited by imperfect information and loss to follow-up in medical records. The results of this systematic review may be affected by the inclusion of lower-quality studies. Second, only six studies provided indications for acromioplasty, and the type of RCTs was not specified; therefore, we could not analyze the effect of acromioplasty for different types of RCTs, and future studies should evaluate the postoperative outcomes of different rotator cuff types. In addition, due to the variety of surgical procedures, the evaluation results may not be reliable. Finally, there are only 2 studies with a follow-up time of more than 2 years and others that did not provide a follow-up which may affect the results of this study. Because the data regarding functional outcomes and complications could not be uniformly compared, a long-term outcome analysis of acromioplasty was not feasible.

## Conclusions

The present systematic review and meta-analysis found that acromioplasty significantly reduced CSA overall, notably in patients with a high preoperative CSA. In addition, the effect on the CSA after lateral acromioplasty is more significant compared to anterolateral acromioplasty. Acromioplasty was not associated with complications during short-term follow-up. Finally, there is a strong need for future studies to clarify the usefulness for acromioplasty at the time of RCR.

## Supplementary Information

**Additional file 1.** PRISMA checklist of the meta-analysis.

## Data Availability

The datasets generated during and/or analyzed during the current study are available from the corresponding author on reasonable request.

## Abbreviations

CSA: Critical shoulder angle
RCTs: Rotator cuff tears
PROs: Patient-reported outcomes
MINORS: The methodological index for non-randomized studies
CI: Confidence interval
